# The effect of images of Michelle Obama’s face on trick-or-treaters’ dietary choices: A randomized control trial

**DOI:** 10.1371/journal.pone.0189693

**Published:** 2018-01-02

**Authors:** Peter M. Aronow, Dean Karlan, Lauren E. Pinson

**Affiliations:** 1 Departments of Political Science and Biostatistics, Yale University, New Haven, CT, United States of America; 2 Department of Finance, Kellogg School of Management, Northwestern University, Evanston, IL, United States of America; 3 Department of Political Science, Yale University, New Haven, CT, United States of America; University of Kansas Medical Center, UNITED STATES

## Abstract

**Objective:**

To evaluate the microfoundations of a personality-inspired public health campaign’s influence on minors.

**Design:**

Multi-year randomized control trial.

**Setting:**

Economics professor’s front porch in New Haven, CT.

**Participants:**

1223 trick-or-treaters in New Haven over three years; on average, 8.5 years old and 53% male (among children whose gender was identifiable).

**Eligibility:**

Trick-or-treaters over the age of three that approached the house.

**Intervention:**

Random assignment to the Michelle Obama side of the porch or the Comparison side of the porch.

**Main outcome measure:**

Selection of fruit over candy.

**Methods:**

Difference-in-means estimates.

**Results:**

We estimate that viewing a photograph of Michelle Obama’s face relative to control conditions caused children to be 19% more likely to choose fruit over candy.

**Conclusions:**

Michelle Obama’s initiative to reduce childhood obesity has influenced children’s dietary preferences. Whether this influence extends beyond Halloween trick-or-treating in New Haven, CT on the porch of an economics professor requires further research.

## Introduction

Obesity remains prevalent among youth in the United States [1] and internationally [2,3]. Based on an analysis of the National Health and Nutrition Examination Survey, 1999–2014, researchers show an estimated 19% increase in the prevalence of Class 1 obesity (low risk obesity, BMI 30.0 to 34.9) in the United States from 1999 to 2014 [4]. Scholars and policymakers have performed a variety of interventions aiming to reduce childhood obesity. While the effectiveness of such interventions varies, meta-analyses suggest school-based interventions may reduce the prevalence of overweight children in the short term [5,6] and compulsory aerobic physical exercise is necessary for efficacy on at least one measure of adiposity [7]. In order to counteract the international epidemic of childhood obesity, scholars must rigorously investigate the effects of relevant policies and initiatives [8,9]. To date, the largest initiatives aimed at reducing childhood obesity have rarely been subject to experimental evaluation.

During her tenure as First Lady of the United States, Michelle Obama has spearheaded one of the largest public health initiatives focused on childhood obesity. In 2010, Obama unveiled her *Let’s Move* Initiative, aimed at fostering a healthy lifestyle and reducing childhood obesity. As the public face of the campaign, Obama urged healthy eating and exercise in a variety of classic and social media venues accessible to minors and their parents, including appearances on *Sesame Street* and *Oprah* and posts of online videos [10]. While the campaign gained publicity, an experimental evaluation of the effects of the entire campaign would not be possible due to the initiative being rolled out across the United States through mass media.

However, we have experimentally assessed the microfoundations of Let’s Move in order to evaluate whether the public health campaign altered dietary decision-making. When children are exposed to the image of a figurehead regularly associated with the public health campaign’s message, the subconscious reminder may reinforce the message. To engage in this investigation, over three years, we ran randomized controlled trials during the evenings of Halloween when trick-or-treating was taking place. Prior experiments provide precedence for experimentally evaluating trick-or-treaters’ decision making [11–12]. By analyzing this series of randomized trials, we are able to provide evidence of the impact of viewing images of Michelle Obama’s face on children’s dietary decision-making.

## Methods

Human Subjects Approval provided by Yale University IRB #1210010995. We use an attention-enhancing technique to understand the impact Michelle Obama’s image has had in shifting not merely attitudes but actual choices children make. We ran the following versions of an experiment during Halloween trick-or-treating in 2012 (n = 165, on average 7.8 years old and 58% male), 2014 (n = 422, on average 8.5 years old and 56% male), and 2015 (n = 636, on average 8.7 years old and 49% male): children were randomly assigned to one of two sides of a porch (In 2012, random assignment was done via drawing pieces of paper from a brown bag; In 2014 and 2015 random assignment was a pseudo-random process via queuing order of children and the speed with which each side completed the last participant). No individual identifying information was collected, and informed consent was neither required by the IRB nor secured. Information on the IRB protocol and experiment was available to any children or parents who asked for such information (fewer than 10 each year asked for such information).

The inclusion criteria was determined merely by individuals that approached the house on Halloween. Children who did not want to wait on line, perhaps due to an objective of maximizing their candy-per-hour productivity rate, did not stop at the household upon sight of the line at the porch. The exclusion criteria was subjectively determined, based on perceived age of the child and their ability to follow instructions to go to one of the tables on the porch. Upon exclusion, the administrator said “Trick or treat” and handed them candy, at which point the child left the porch.

One side of the porch–denoted *Obama*–had a large photo of Michelle Obama in front of the children. The other side–denoted *Comparison*–had a photo of Ann Romney (2012), a photo of Hillary Clinton (2014, 2015), or no photo (2014, 2015). (See S1 Table for the disaggregation of comparison groups.) Other posters were taped to the wall that said either “Obama” or “Romney”/“Clinton” on the respective sides of the porch. In 2014, we primed the trick-or-treater by pointing to the photo and asking if they knew who that was; in 2015, trick-or-treaters were individually randomly assigned (using a pre-coded Excel spreadsheet) to receive or not receive the additional priming. We pool our analysis, irrespective of this additional source of variation. Prior to being led to a side of the porch, children would be queued at the stairs leading up to the porch. Some children likely could see each side of the porch, although many undoubtedly did not due to pathway-blocking chairs, chaos, and darkness.

At both sides of the porch, children were asked their age and whether they would prefer to receive fruit (a box of raisins) or candy (a more typical small packaged piece of name-brand chocolate such as Snickers or Milky Ways). Each child was given the option (fruit or candy) that they requested. We recorded each child’s reported age, gender (when identifiable), and choice of fruit or candy. The experimental set-up allows us to measure what proportion of children chose fruit instead of candy when in the presence of an image of Michelle Obama’s face, as well as the proportion of children who chose fruit instead of candy when not in the presence of an image of Michelle Obama’s face.

The experiment was conducted in the East Rock neighborhood of New Haven, CT. The East Rock neighborhood is one mile from the Yale University campus, and contains many single family homes owned by Yale faculty, as well as some multi-family homes in which many graduate students live. There are also low income neighborhoods within a mile of this neighborhood. Furthermore, due to the high level of activity during Halloween, many families drive from further away in order to trick-or-treat in this neighborhood. From an earlier study [11], we know that the population that trick-or-treats in this neighborhood is heavily liberal politically, with about 79% of children informally "voting" for Obama over McCain in 2008 and 82% for Obama over Romney in 2012.

For analysis, we use the difference-in-means estimates for each year. (We had missing outcome data on one 12 year old, female participant in 2012; this participant is dropped from analysis. Our findings are substantively unchanged by imputing either a fruit or candy outcome for the participant.) To compute the pooled estimates, we aggregate across years using inverse probability of treatment weighting. For all estimates, we form Wald-type confidence intervals using a normal approximation-based estimation using heteroskedasticity-robust standard errors as calculated in R 3.2.3.

## Results and conclusions

**Fig 1:**
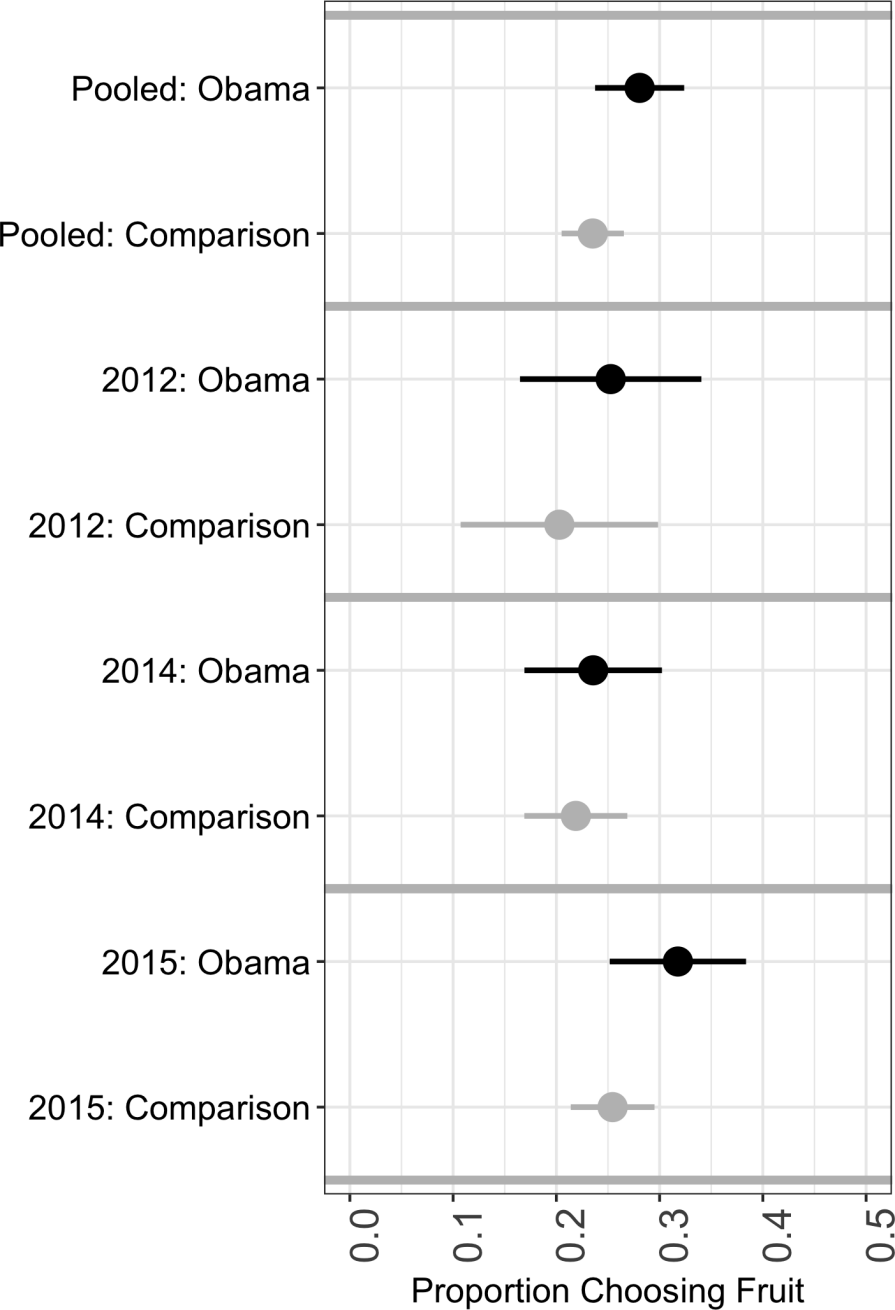
Estimates of mean preferences for fruit over candy among trick-or-treaters, 95% confidence intervals.

As shown in Fig 1, we estimate that 28.1% (95% CI 23.8–32.4%) of participants in the Obama treatment select fruit instead of candy, in contrast to only 23.5% (20.5–26.5%) of participants in the pooled comparison group. The difference is statistically significant at the 0.10 level (two-sided *p* = 0.091). We present the estimates for each year in Fig 1.

Sample selection issues limit the generalizability of the experimental findings. For example, this experiment may not allow us to extrapolate the influence of viewing Michelle Obama’s face on children’s dietary decision making outside of an economics professor’s front porch in New Haven on Halloween night. Based on the location of the study, the likely socioeconomic status and political leanings of the children’s parents make the findings of this study not generalizable beyond trick-or-treaters in the New Haven area. In particular, this is a strongly Democratic neighborhood, and if children in Democratic households are either more aware of Michelle Obama’s campaign or more favorably disposed to it, we would generate an overestimate of the treatment effect of her campaign on the general population of those in the United States.

Furthermore, replication of this study may be hindered by variation across years, some inevitable, and some possible to study further. Hillary Clinton, for example, may generate a different effect before or after the 2016 election, because of changes in opinion, whether favorable or not, generated from the 2016 campaign. Furthermore, the method employed for priming individuals each year differed, and further research may benefit from examining how such priming affects the treatment effect noted.

In addition, since we conducted the experiment on three Halloweens at the same location, repeat participation and memory of prior participation was possible. This too may limit the generalizability of our pooled findings to settings outside of a series of repeated randomized controlled trials on an economics professor’s front porch.

Furthermore, since we ran the experiment on a day where candy is readily available, the influence on children’s dietary preference for fruit instead of candy may differ from other days of the year; for instance, perhaps children are more willing to choose fruit since it is unique for the holiday, or children are less willing to choose fruit because they are under the influence of sugar consumption.

## Supporting information

S1 Table Disaggregation of data. (DOCX)
