# Supplementary material for: The effect of images of Michelle Obama’s face on trick-or-treaters’ dietary choices: A randomized control trial
Source: PLoS One. 2018 Jan 2;13(1):e0189693. doi: 10.1371/journal.pone.0189693 (PMC5749710; doi:10.1371/journal.pone.0189693)
Supplement: S1 Table — (DOCX) [file pone.0189693.s001.docx]

**S1 Table: Disaggregation of data.**

| **Year** | **Assignment** | **N** | **Mean (SE)** |
| --- | --- | --- | --- |
| **2012** | Obama | 95 | .25 (.04) |
|  | Romney | 69 | .20 (.05) |
| **2014** | Obama | 157 | .24 (.03) |
|  | Clinton | 130 | .27 (.04) |
|  | No photo | 135 | .17 (.03) |
| **2015** | Obama | 192 | .32 (.03) |
|  | Clinton | 207 | .27 (.03) |
|  | No photo | 237 | .24 (.03) |
